# Aphid populations showing differential levels of virulence on *Capsicum* accessions

**DOI:** 10.1111/1744-7917.12648

**Published:** 2018-12-06

**Authors:** Mengjing Sun, Roeland E. Voorrips, Ben Vosman

**Affiliations:** ^1^ Plant Breeding Wageningen University & Research Wageningen The Netherlands

**Keywords:** callose deposition, EPG, plant–aphid interaction, plant immunity, ROS accumulation, virulence

## Abstract

The green peach aphid, *Myzus persicae*, is one of the most threatening pests in pepper cultivation and growers would benefit from resistant varieties. Previously, we identified two *Capsicum* accessions as susceptible and three as resistant to *M. persicae* using an aphid population originating from the Netherlands (NL). Later on we identified an aphid population originating from a different geographical region (Switserland, SW) that was virulent on all tested *Capsicum* accessions. The objective of the current work is to describe in detail different aspects of the interaction between two aphid populations and two selected *Capsicum* accessions (one that was susceptible [PB2013046] and one that was resistant [PB2013071] to population NL), including biochemical processes involved. Electrical penetration graph (EPG) recordings showed similar feeding activities for both aphid populations on PB2013046. On accession PB2013071 the aphid population SW was able to devote significantly more time to phloem ingestion than population NL. We also studied plant defense response and found that plants of accession PB2013046 could not induce an accumulation of reactive oxygen species and callose formation after infestation with either aphid population. However, plants of PB2013071 induced a stronger defense response after infestation by population NL than after infestation by population SW. Based on these results, population SW of *M. persicae* seems to have overcome the resistance of PB2013071 that prevented feeding of aphids from NL population. The potential mechanism by which SW population overcomes the resistance is discussed.

## Introduction

Aphids are among the most important plant pests worldwide, damaging crops directly by feeding from the phloem and indirectly by transmitting many harmful viruses (Dixon, 1977; Powell *et al*., 2006). The generalist green peach aphid, *Myzus persicae*, is one of the most important pest insects in pepper crops (*Capsicum* spp.), causing chlorosis, leaf defoliation, flower and fruit abortion (Blackman & Eastop, 2000). Many pepper viruses are mainly vectored by *M. persicae*, including Pepper mottle virus, Pepper severe mosaic virus and Pepper yellow mosaic virus (Black *et al*., 1991; Kenyon *et al*., 2014). Chemical pesticides have been widely used to control aphids. However, due to the long‐time use of these chemicals, more and more species (and populations) of aphids are reported to be developing resistance to pesticides (Wang *et al*., 2002; Cheng *et al*., 2004; Bass *et al*., 2014). With increasing concern about the negative environmental impact of insecticides, host plant resistance is commonly seen as a desirable goal in plant breeding and is projected to play an indispensable role in integrated pest management (Broekgaarden *et al*., 2011). In many cases, resistance factors like Quantitative Trait Loci (QTLs) or genes controlling plant resistance have been successfully used in breeding programs, such as the resistance in lettuce to the black currant‐lettuce aphid *Nasonovia ribisnigri* (Eenink *et al*., 1982), the resistance in wheat to the Russian wheat aphid *Diuraphis noxia* (Cleveland *et al*., 2003), the resistance in soybean to soybean aphid *Aphis glycines* (Wu *et al*., 2004) and the resistance in melon to cotton aphid *Aphis gossypii* (Pitrat & Lecoq, 1980). One type of plant resistance mechanism was hypothesized to work according to the gene‐for‐gene principle, which means that a resistance gene (*R* gene) in the resistant plant recognizes an effector secreted by the aphid and then activates defense responses against the attacking aphid (Stotz *et al*., 1999; Kessler & Baldwin, 2002). Later on the more comprehensive zigzag model was developed (Jones & Dangl, 2006; Smith & Boyko, 2007; Yates & Michel, 2018). During aphid infestation, plants can recognize conserved molecules (known as pathogen or herbivore‐associated molecular patterns or PAMPs/HAMPS) by pattern recognition receptors (PRR) and activate PAMP‐triggered immunity (PTI) (Jones & Dangl, 2006; Smith & Boyko, 2007). In order to colonize plants, aphids may secrete effectors to prevent the plant defense response, which is known as effector‐triggered susceptibility (ETS) (Rodriguez & Bos, 2013; Elzinga *et al*., 2014). At their turn plants may respond with the production of R proteins that are able to recognize effectors, leading to effector‐triggered immunity (ETI) (Hogenhout & Bos, 2011; Jaouannet *et al*., 2014). Both PTI and ETI result in an incompatible plant–aphid interaction (Tsuda & Katagiri, 2010). The incompatible interaction between host and insect may be observed as a microscopic hypersensitive response in the host plant after insect infestation, involving phloem protein plugging (Tjallingii, 2006; Medina‐Ortega & Walker, 2015), callose deposition (Villada *et al*., 2009; Luna *et al*., 2011), and/or accumulation of reactive oxygen species (ROS) (Moloi & van der Westhuizen, 2006; Villada *et al*., 2009; Lei *et al*., 2014). Phloem protein plugging is a fast process, which has been best studied in legumes, involving forisomes (Peters *et al*., 2006). So far there is only limited information on protein plugging of sieve elements in other species (Knoblauch *et al*., 2014; Garzo *et al*., 2018). The deposition of callose, a β‐1,3‐glucan, has been reported as an important and long‐lasting reaction to wounding, pathogen infection and insect infestation (Stone & Clarke, 1992; Donofrio & Delaney, 2001; van der Westhuizen *et al*., 2002; Hao *et al*., 2008). Phloem protein plugging and callose deposition induced by phloem‐feeding insects are triggered by an influx of calcium. They prevent the uptake of sieve‐tube sap by the insect and is suggested to be a resistance factor against several insects (Van der Westhuizen *et al*., 1998; Liu *et al*., 2017; Sun *et al*., 2018). The accumulation of ROS is an earlier and faster reaction than callose deposition after pathogen or insect attack (Piedras *et al*., 1998; Miller *et al*., 2009). ROS accumulation is believed to play an important role in plant resistance to invading aphids (Moloi & van der Westhuizen, 2006; Kerchev *et al*., 2012; Shoala *et al*., 2018). Not only does it protect plants directly (Liu *et al*., 2010), it also acts as signal to activate downstream defense enzymes (Moloi & van der Westhuizen, 2006; Kuśnierczyk *et al*., 2008). The incompatible host–aphid interaction also can be detected by monitoring aphid probing and feeding behavior using the electrical penetration graph (EPG) technique (Alvarez *et al*., 2006; Chandran *et al*., 2013). The EPG technique provides information about the aphid's activity on the plant through different waveforms (Tjallingii, 1988; Tjallingii *et al*., 2010) and these waveforms have been used to deduce the physical location of resistance factors encountered by aphids (Alvarez *et al*., 2006; Khan *et al*., 2015).

Although breeding resistant varieties is a promising method to manage aphid populations, one challenge is to prevent the evolution of new aphid populations which can overcome the resistance (Haley *et al*., 2004; Hill *et al*., 2010; ten Broeke *et al*., 2013a). An aphid population that can overcome host resistance is called a virulent population. Virulent populations are often found with specialist aphids such as *Diuraphis noxia* (Haley *et al*., 2004), *A. glycines* (Kim *et al*., 2008), and *A. pisum* (Kanvil *et al*., 2014). For generalist aphids, there are only a few reports showing that certain populations of *Macrosiphum euphorbiae* (Hebert *et al*., 2007; Pallipparambil *et al*., 2010), *A. gossypii* (Lombaert *et al*., 2009), and *M. persicae* (Cabrera‐Brandt *et al*., 2015) can overcome or partially overcome crop resistance. To prevent the emergence of virulent or semivirulent aphid populations it is important to understand how they overcome the resistance. Previous studies which revealed the existence of virulent aphid populations mostly payed attention to the variation in aphid behavior on resistant plants. A more detailed study on the interaction, which involves not only aphid behavior but also constitutive and induced plant resistance mechanisms, may help to understand the mechanism by which a virulent aphid population overcomes resistance.

Recently, we identified *Capsicum* accessions susceptible and resistant to a *M. persicae* population from the Netherlands (Sun *et al*., 2018). These accessions were also challenged with a *M. persicae* population originating from a different geographical region (Switzerland). Aphid feeding activity and plant defense responses were studied in the various aphid–plant combinations in order to elucidate in detail different aspects of the interaction between the pepper accessions and the two aphid populations.

## Materials and methods

### Plant materials

The plant materials used are *C. baccatum* accessions (PB2013046, PB2012022, PB2013062 and PB2013071, obtained from the collection of Wageningen University & Research, NL) and a *C. annuum* accession (CGN19226, obtained from the Centre for Genetic Resources, NL). About 2 weeks after sowing, plants were transplanted into 14 cm pots with potting compost and grown in a standard greenhouse at 19–21 °C, 60%–70% relative humidity and a 16–8 h light–dark photoperiod at Wageningen University & Research, NL. Plants were watered every other day. No insect control was applied during growth and testing of the plants.

### Aphid populations

Two populations of *M. persicae* were used in this study. One population was collected in the Netherlands in the 1980s and reared for many years on Chinese cabbage (*Brassica rapa* L. ssp. *pekinensis* cv. Granaat) at Wageningen University & Research, NL. The other population originates from Switzerland where it was collected in 1982. It was reared on peas until 2013, when it was transferred to *C. annuum*. The populations are referred to as NL and SW, respectively. We refer to them as populations, as it is unclear if they were started from one single aphid. They may in fact be two different clones. For the experiments discussed here, both populations were reared since 2015 on *C. baccatum* accession PB2013046 under the same conditions as used for growing of the pepper plants.

### Evaluation of aphid performance by a clip cage test

The evaluations were performed in 2016 in a greenhouse of Wageningen University & Research, NL, when the plants were seven weeks old and still in the vegetative stage. Five plants of each accession were used per aphid population. All plants were randomized in one greenhouse compartment. Each plant received three clip cages (25 mm diameter), containing five 1‐d‐old nymphs from either the NL or SW population. The 1‐d‐old nymphs were produced by putting adult aphids on a clean leaf for 24 h and collecting all nymphs produced during that period, which were then used for infestation. After 12 d the number of surviving and dead aphids as well as new nymphs produced in each clip cage were counted. Statistical analysis was carried out as described previously (Sun *et al*., 2018). The observations from the three clip cages per plant were combined. Aphid survival was determined by dividing the number of living aphids by the total number of original aphids (dead and alive) that were found back in the clip cage. The number of next generation nymphs per original aphid was calculated by dividing the number of next generation aphids by the average number of living aphids present in the clip‐cage, which was calculated as (2 × living aphids + dead aphids)/2. In this formula we assume that dead original aphids contributed to the offspring during half of their life. Given that some aphids were able to escape from the clip cages because of the uneven leaf surface, data from clip cages with less than four aphids (dead and alive) were not included in the analysis. For statistical analysis data were transformed to stabilize the residual variance: survival as arcsin[sqrt(*x*)] and nymphs produced per average living adult as sqrt(*x*). Significance of accession effects (five tested accessions) was evaluated using ANOVA and the LSD test (*P* < 0.05) was used to assess pairwise differences between accessions, and between the two aphid populations using the *t*‐test (*P* < 0.05).

### Monitoring of aphid probing and feeding behavior

The Electrical Penetration Graph (EPG) technique was used to monitor probing and feeding behavior of the two aphid populations on *C. baccatum* accessions PB2013071 and PB2013046, which were resistant and susceptible to the aphids of the NL population, respectively. Seven‐week‐old plants were probed with one adult aphid per plant placed on the abaxial side of the second fully expanded leaf from the top. For each recording a new aphid and plant were used. The EPG setup was as described by Alvarez *et al*. (2013). EPG recordings lasted for 6 h and were carried out under constant light and at a temperature of 20 ± 2 °C. We made 14 recordings (one per aphid) with each population on accession PB2013071, and 13 recordings with each population on accession PB2013046, after removing incomplete recordings because of aphid escape, respectively. The Stylet+ analysis software version 1.20 (http://www.epgsystems.eu/) was used to convert EPG recordings into different waveforms. EPG parameters were calculated online using EPG‐Calc 6.1.3 (Giordanengo, 2014). When a waveform was not produced, its duration was set to 0 (zero). *t*‐tests were used to determine the significance of both the differences between the accessions treated by the same aphid population and the differences between two aphid populations feeding on the same accession. Parameters that represent a fraction (such as parameter “% of *E*1 to *E*”) were transformed as arcsin[sqrt(*x*)] to stabilize variances. Other parameters were transformed to Ln(*x*+1) if needed. All the *t*‐tests were done in R v3.4.1 (https://www.R-project.org/) with default packages.

### DAB staining for ROS accumulation

DAB (3,3’‐Diaminobenzidine) staining was performed according to the protocol of Daudi and O'Brien (2012) on plants of the accessions PB2013071 and PB2013046 after infestation with the two aphid populations. Seven‐week‐old plants received three clip cages containing 15 randomly selected wingless adult aphids per cage or three empty clip cages. Per accession we used four biological replicates (four plants) per aphid population. Leaf disks were collected from the clip cage areas after 6 h of aphid infestation, and disks under empty clip cages were collected at the same time for reference. Feeding aphids were removed from the leaves with a brush, and disks were then placed in 1 mg/mL 3,3’‐diaminobenzidine (DAB)‐HCl (Sigma‐Aldrich, USA) followed by vacuum infiltration for 20 min. After that, the disks were gently shaken and incubated overnight at room temperature in the dark. The next day they were cleaned with 96% ethanol in a 65 °C water bath for 3 h or in boiling water for 30–40 min. Ethanol was replaced when needed. After chlorophyll was removed, samples were washed in 30% ethanol and then mounted on glass slides with 30% glycerol. The presence of ROS was manifested by brown polymerized deposits. Photos were taken using a Zeiss Axiophoto digital imaging microscope (Carl Zeiss AG, Germany).

### Callose deposition

Histological analysis of *in situ* callose deposition was carried out on accessions PB2013071 and PB2013046, infested with two aphid populations when plants were seven weeks old. Plants received either an empty clip cage or a cage with aphids. Three leaves each with one clip cage from three independent biological replicates per treatment were collected 24 h after the start of aphid infestation. Fifteen randomly selected wingless adult aphids were used in one clip cage. Leaf disks under an empty clip cage were collected after 24 h and used as reference. Aphids on disks were gently brushed away, and then leaf disks were washed and stained according to (Kissoudis *et al*., 2016; Sun *et al*., 2018). Samples were subsequently mounted on glass slides with 50% glycerol. Callose fluorescence was observed under UV light and photos were taken using the Zeiss Axiophoto digital imaging microscope (Carl Zeiss AG, Germany). The number of fluorescent callose spots in each disk was counted. For statistical analysis, the significance of differences in the average number of callose spots from three treatments (NL population, SW population and uninfested reference) was evaluated using ANOVA with the LSD test (*P* < 0.05).

## Results

### Aphid performance

**Figure 1:**
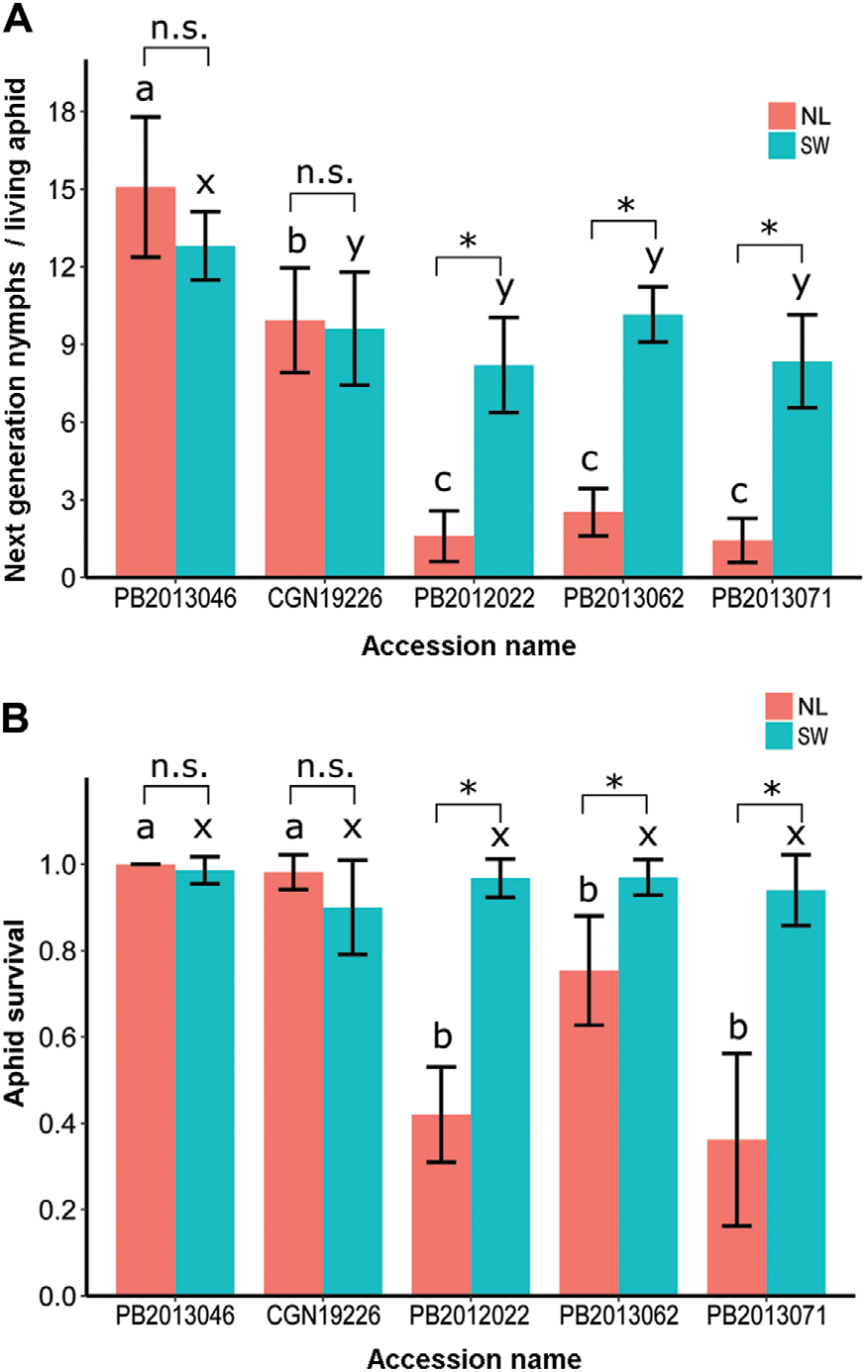
Performance of *Myzus persicae* populations NL and SW on five pepper accessions. (A) Average number of next generation nymphs produced per living adult after 12 d. (B) The fraction of aphids initially put on a plant that survived 12 d. Each bar represents the mean values ± SD of five plants per accession. Within each panel, pink bars labeled with the same letter (a, b, or c) are not significantly different from each other and similar for the blue bars (*x* and *y*), (LSD test, *P* = 0.05). Within each set of two bars a significant difference is indicated by * and a nonsignificant one by n.s. (*t*‐test, *P* = 0.05).

The aphid populations NL and SW, which were collected in the Netherlands and Switzerland respectively, can survive and reproduce well on accessions PB2013046 and CGN19226 (Fig. 1, Table S1). More than 90% of the 1‐d‐old nymphs of each population survived and developed into adults, and on average each aphid produced more than 10 offspring after turning into adults. However, reproduction on accession PB2013046 was significantly higher than on accession CGN19226 for both aphid populations (Fig. 1A, LSD, *P* < 0.05, Table S1).

On the other three accessions (PB2012022, PB2013062, and PB2013071), the population of NL aphids produced fewer next‐generation nymphs than on the accessions PB2013046 and CGN19226, whereas SW aphids produced significantly fewer nymphs than on PB2013046, but not compared with CGN19226 (Fig. 1A, LSD, *P* < 0.05, Table S1). Moreover, NL aphids on these three accessions produced significantly fewer next generation nymphs than SW aphids (Fig. 1A, *t*‐test, *P* < 0.05, Table S1). Aphids of the NL population showed a significantly lower survival on these three accessions than on the other two accessions (PB2013046 and CGN19226) (Fig. 1B, LSD, *P* < 0.05, Table S1), while aphids of SW population showed a similar survival level on all accessions.

### EPG analysis

The EPG technique was used to study aphid feeding behavior on the pepper accessions PB2013071 and PB2013046 (resistant and susceptible to the NL population, respectively) using both aphid populations. Tables 1 and S2 show the results for some EPG parameters.

**Table 1:** EPG parameters (mean value ± standard error) measured for *M. persicae* populations NL and SW on accessions PB2013071 and PB2013046. The following codes are used: no‐penetration period (NP), intercellular apoplastic stylet pathway (C), derailed stylet mechanics (F), xylem ingestion (G), phloem phase (E), phloem salivation at the beginning of the phloem phase (E1), and passive phloem ingestion (E2). Time spent in each phase is given in minutes. The number of recordings for each aphid population–accession combination is indicated below the accession number

	Parameter value				*P* value from *t*‐test			
	Population NL		Population SW		Population NL	Population SW	PB2013071	PB2013046
	PB2013071 (*n* = 14)	PB2013046 (*n* = 13)	PB2013071 (*n* = 14)	PB2013046 (*n* = 13)	PB2013071 VS PB2013046	PB2013071 VS PB2013046	Population NL VS Population SW	Population NL VS Population SW
Number of probes	13.8±2.4	11.2±2.5	10.4±2.7	12.0±2.8	0.2687	0.5956	0.1948	0.7673
Total duration of probes	342.9±3.2	348.2±2.5	333.7±6.1	341.7±4.5	0.1129	0.5038	0.3994	0.2880
Number of NP	12.9±2.4	10.2±2.5	9.4±2.7	11.1±2.8	0.2542	0.5785	0.1830	0.7470
Total duration of NP	16.9±3.2	11.4±2.6	16.5±3.8	17.6±4.6	0.1125	0.8793	0.9362	0.3222
Number of C	26.3±2.5	16.7±2.9	15.8±3.1	14.5±2.8	0.0025	0.7041	0.0030	0.4814
Total duration of C	162.1±6.3	122.5±7.5	127.7±8.5	105.7±7.6	0.0482	0.4003	0.1458	0.4588
Number of F	3.2±1.8	2.9±1.5	0.5±0.8	1.2±1.2	0.7945	0.1162	0.0090	0.0422
Total duration of F	64.3±8.6	111.9±7.8	48.1±8.8	77.8±9.3	0.0823	0.3674	0.5846	0.2625
Total duration of G	45.3±5.7	19.0±4.6	9.4±4.2	12.4±5.1	0.0199	0.7306	0.0018	0.4841
Time to first G	176.0±10.2	264.8±10.2	334.7±8.1	325.3±8.6	0.0357	0.7341	0.0000	0.1022
Total duration of E	71.2±6.6	94.8±9.2	156.7±10.4	145.8±10.3	0.3830	0.7995	0.0178	0.1913
Time to first E	63.9±6.8	154.6±9.4	48.0±6.3	121.1±10.3	0.0042	0.0329	0.3481	0.3893
Total duration of E1	71.2±6.6	19.8±4.7	75.1±8.1	20.2±3.3	0.0007	0.0111	0.8566	0.9541
Number of single E1	9.1±1.9	2.5±1.8	4.6±1.9	1.2±1.0	0.0000	0.0045	0.0026	0.0817
Total duration of single E1	69.9±6.6	13.5±4.0	42.6±6.7	5.5±2.6	0.0003	0.0117	0.1236	0.1084
Number of E1 followed by E2	0.9±1.6	0.7±0.8	2.0±1.4	1.8±1.0	0.8265	0.3123	0.2346	0.0062
Total duration of E1 followed by E2	1.3±2.0	6.3±3.2	32.5±7.5	14.7±3.3	0.0151	0.2896	0.0717	0.0518
% of E1 to E	99.9±0.6	50.4±6.7	67.7±6.4	30.1±5.7	0.0020	0.0160	0.0149	0.2034
Total duration of E2	0.1±0.4	74.9±9.5	81.6±10.7	125.6±10.4	0.0107	0.0500	0.0257	0.2038
Time to first E2	340.4±7.8	284.8±9.5	231.9±11.0	187.7±10.4	0.0108	0.3352	0.0092	0.0207
Total duration of sE2†	0.0±0.0	74.8±9.5	76.3±10.4	124.5±10.4	0.0000	0.0298	0.0263	0.2153
Time to first sE2†	360.0±0.0	284.9±9.5	266.0±11.0	195.3±10.7	0.0044	0.1377	0.0152	0.0371
Number of potential drops	152.9±6.7	69.9±6.2	106.9±7.5	65.4±6.1	0.0000	0.0390	0.0296	0.7674

^†^sE2 represents sustained E2, phloem ingestion lasting for longer than 10 min.

### Comparison between pepper accessions

For both aphid populations many differences were observed between the two accessions during the phloem feeding phase. More time was spent on phloem salivation and much less time on phloem ingestion by aphids on accession PB2013071 than on accession PB2013046. The time until the first phloem event was shorter for aphids on accession PB2013071 than for those on accession PB2013046. In addition, the total number of potential drops (individual cell punctures) for both aphid populations was higher on accession PB2013071 than on accession PB2013046.

For some parameters, the aphids of the NL population showed clear and significant differences in performance on the two accessions while the aphids of the SW population did not show a significant difference. These included the total time spent in the intercellular apoplastic pathway phase, the number of this pathway phases and the total time spent on xylem sap ingestion (all larger on accession PB2013071 than on accession PB2013046) and the time until first xylem probing (shorter on accession PB2013071).

### Comparison between aphid populations

Aphids of the SW population were more successful than the aphids of the NL population when feeding on accession PB2013071, which is also more resistant to the NL population in terms of survival and reproduction. Although no significant differences between both aphid populations were detected in the overall duration of phloem salivation and the time until first phloem event, aphids of SW population spent much more time on phloem ingestion and needed less time until the first phloem ingestion compared to aphids of the NL population. The SW population also had a smaller number of intercellular apoplastic pathway phases, derailed stylets, xylem ingestion, and potential drops compared with the NL population.

While on accession PB2013046 only minor differences between the two aphid populations were observed: aphids of the NL population had more penetration difficulties (higher number of F) and needed a longer time until the first phloem ingestion compared with aphids of the SW population.

### ROS accumulation

**Figure 2:**
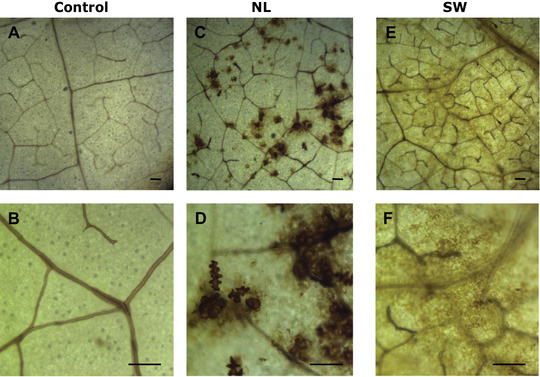
ROS accumulation in leaves of pepper accession PB2013071 in response to *M. persicae* populations NL and SW. DAB staining was used to show ROS accumulation after 6 h in leaves under empty clip cages (A, B) and under clip cages after a 6 h infestation with aphids of the NL (C, D) or SW (E, F) population. Bars = 200 μm. Photos B, D, F were taken with higher magnification on the same leaf disk than photos A, C, E, respectively.

To investigate possible differences in the accumulation of reactive oxygen species (ROS) in plants when aphids of the NL or SW population were present, leaf disks (under the clip cages) where aphids had been feeding for 6 h were collected and stained for ROS accumulation. No ROS accumulation was seen in leaf disks from the accession PB2013046 with either aphid population or without aphids (Fig. S1). On accession PB2013071 (resistant to the NL population), dark staining was observed on leaf disks infested with aphids from the NL population and stained spots were mostly distributed along leaf veins. Conversely, only a very weak staining signal was seen on leaves of this accession infested with SW aphids (Fig. 2) and no staining was observed at all on leaf disks under empty clip cages.

### Callose deposition

**Figure 3:**
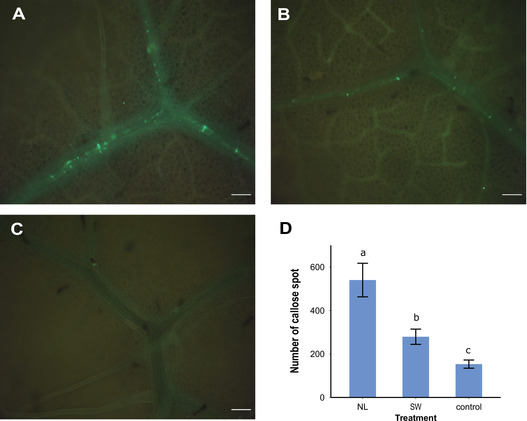
Callose deposition induced by *M. persicae* populations NL and SW on accession PB2013071. (A–C) Callose depositions in pepper leaves under clip cages after a 24h infestation with aphids of the NL (A) or SW (B) population and under an empty cage (C). (D) Shows the number of callose spots counted per leaf disk under the clip cage area. Bars represent means ± SD. Different letters indicate statistically significant differences between treatments (LSD‐test at *P* < 0.05).

The formation of callose was examined to explore differences in defense after infestation with aphids of the NL or SW population on accessions PB2013071 and PB2013046 (resistant and susceptible to the NL population, respectively). No callose signal was detected in plants of the accession PB2013046 with either aphid population or in leaf disks without aphids infestation (results not shown). A clear callose signal was found in the vascular system of plants of the accession PB2013071 after 24 h of infestation with either aphid population (Fig. 3A and B). More fluorescent signal was detected in leaf disks infested with aphids of the NL population compared to the SW population (Fig. 3D, LSD, *P* < 0.05).

## Discussion

### Resistance in accession PB2013071 seems to be overcome by aphids of the SW population

The five accessions can be classified into resistant or susceptible based on differences in the performance of the aphid population from the Netherlands (NL) for both parameters used: survival of the original nymphs and the number of next generation nymphs produced. When using the SW population on plants of the three accessions resistant to the NL population (PB2012022, PB2013062, and PB2013071), we found that aphids of the SW population always had a higher survival and produced more offspring than those of the NL population. This difference between the two aphid populations was not seen on plants of accessions PB2013046 and CGN19226, on which both aphid populations behaved the same. Similar results were obtained in other studies involving other aphids and host plants; different populations of an aphid species performed differently on resistant, but not on susceptible plants (Pallipparambil *et al*., 2010; ten Broeke *et al*., 2013a,b). During EPG recordings, many differences were observed in the feeding of aphids from the two populations on accession PB2013071 that is resistant to the NL population, and these differences were apparent in all phases except the nonprobing phase, although not for all parameters. The most important difference between the two populations was seen during the phloem feeding phase. Both aphid populations were able to start phloem ingestion, but only aphids of the SW population were able to continue feeding for a prolonged time, resulting in a large difference in the length of the E2 phase. Probably because of successful phloem feeding, aphids of the SW population were able to propagate on accession PB2013071, as was shown by the performance experiment. In contrast, for aphids of the NL population it was almost impossible to take up phloem sap. These aphids often switched to xylem ingestion, perhaps to prevent starvation (Helden & Tjallingii, 1993; Crompton & Ode, 2010). Compared to aphids of the SW population, an attack by aphids of the NL population induced a stronger defense response in accession PB2013071. This induction was accompanied by a clearer ROS accumulation and more callose deposition. As one of the functions proposed for ROS is that it acts as a local toxin and discourages the attacker (Chen & Schopfer, 1999; Liu *et al*., 2010), it might be expected that strong ROS accumulation in resistant pepper leaves is induced directly in the phloem vessels, and this is indeed suggested by the distribution of stained spots along leaf veins in our case. Also in the leaves of accession PB2013071 more callose deposits were found upon infestation with NL aphids than with SW aphids. More callose deposition may lead to more serious occlusion of the phloem vessels and cause more difficulties to aphids during prolonged feeding (Hao *et al*., 2008; Sun *et al*., 2018). However, the fast plant reaction that prevents NL aphids from feeding might be caused by phloem proteins (Tjallingii, 2006; Furch *et al*., 2009). Coagulation of phloem proteins may cause the occlusion (plugging) of sieve elements and the aphid food canal (Garzo *et al*., 2018; Peng & Walker, 2018). Further experiments are needed to elucidate what is going on during this fast response in pepper‐aphid interaction. Based on all these data presented in our study, the resistance mechanism in accession PB2013071 seems to be much less effective against the SW population than against the NL population. Compared to aphids of the NL population, those of the SW population were able to initiate sustained phloem ingestion and only induced a mild defense response, suggesting that the aphids of the SW population are (semi)virulent on PB2013071 and have for a large part overcome the resistance. Such differences in virulence between populations were also reported for other aphid species and on other host plants (Tolmay *et al*., 2007; Lombaert *et al*., 2009; ten Broeke *et al*., 2013b). However, in our case population SW can only be termed semivirulent because accession PB2013071 still shows some residual resistance to the SW aphids.

### Pepper accession PB2013071 shows residual resistance to the SW population

The EPG analysis revealed that aphids of the SW population to some extent experienced difficulties in taking up the phloem sap on the plants of accession PB2013071. The phloem salivation periods were longer and the phloem uptake periods were shorter on accession PB2013071 than on accession PB2013046. Differences were also detected in the level of ROS accumulation and callose deposition between both accessions after the infestation with the SW aphid population. No ROS accumulation and no callose deposits were found in the leaves of accession PB2013046 after infestation, whereas weak signals were clearly present in the resistant accession. These observations suggest that there still are resistance components in PB2013071 showing some residual effectivity against the SW population. Similar studies by others show that virulent aphids or pathogens are sometimes not able to overcome resistance completely and show a reduced virulence, therefore they are called as semivirulent (Stewart *et al*., 2003; Tan *et al*., 2008; Humphries *et al*., 2013; Humphries *et al*., 2016).

### The interaction between two aphid populations and pepper accession PB2013071

**Figure 4:**
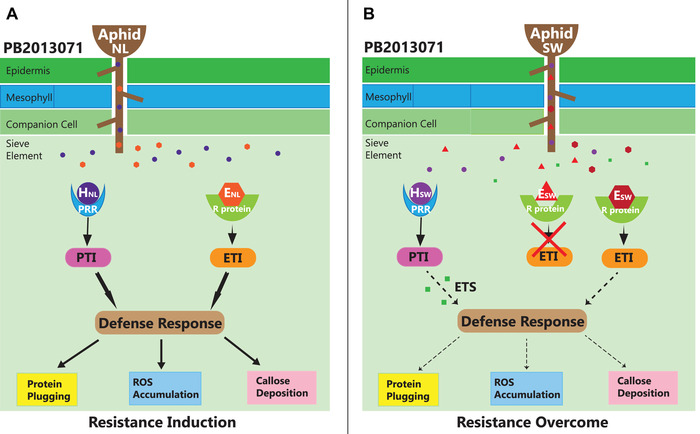
Model explaining different interactions with pepper accession PB2013071 induced by two different *M. persicae* populations. Aphids of the NL and SW populations use their stylets to ingest phloem sap of accession PB2013071. Saliva is secreted during probing and feeding. The herbivore‐associated molecular patterns (HAMPs, such as H_NL_ and H_SW_) from the saliva of both aphid populations might be recognized by pattern recognition receptors (PRRs) from accession PB2013071, and induce PAMP‐triggered immunity (PTI). The PTI involves a defense response, which may include plugging of the phloem by proteins, ROS accumulation and callose deposition. To circumvent/suppress plant defenses, aphids may produce specific effectors resulting in effector‐triggered susceptibility (ETS). In turn, the plant may respond by producing specific resistance (R) proteins that recognize the effector (such as E_NL_ and E_SW_) of the aphid, resulting in effector‐triggered immunity (ETI). The defense responses involving in ETI normally overlap with those in PTI. (A) Resistance of accession PB2013071 to NL aphids might be caused by induction of PTI, due to recognition of H_NL_, or by induction of ETI, due to recognition of E_NL_. (B) Accession PB2013071 is only partially resistant to SW aphids because both PTI and ETI are (at least partially) suppressed, perhaps due to ETS triggered by some SW effectors, or failure of R proteins to recognize SW effectors, or suppression of ETI. H and circles indicate HAMPs; E and polygons (triangles, squares and hexagons) indicate effectors. Black arrows and dashed arrows mean induced and (partially) suppressed responses of PB2013071, respectively.

The interaction between aphids and their host plants is often hypothesized to follow the gene‐for‐gene principle (Flor, 1971; Stotz *et al*., 1999; Kessler & Baldwin, 2002), which has been developed into the more comprehensive zigzag model (Jones & Dangl, 2006; Smith & Boyko, 2007; Yates & Michel, 2018). When aphids attack a plant, herbivore‐associated molecular patterns (HAMPs) from aphid saliva might be recognized by pattern recognition receptors (PRRs), causing PAMP‐triggered immunity (PTI) (Hogenhout & Bos, 2011). Insects may develop effectors that suppress PTI which is called effector‐triggered susceptibility (ETS) (Rodriguez & Bos, 2013; Elzinga *et al*., 2014; Wang *et al*., 2015). In their turn, plants may develop R proteins that recognize effectors in the saliva of the aphids and thus through effector‐triggered immunity (ETI) restore resistance (Bos *et al*., 2010; Chaudhary *et al*., 2014). If we apply this model to our system we may hypothesize that accession PB2013071 is resistant to the NL aphid population through PTI or ETI (Fig. 4A) while it is partially susceptible to the SW aphids because PTI is suppressed by effectors from SW aphids (Pitino & Hogenhout, 2013; Rodriguez *et al*., 2017), ETI is not activated because of lack of effectors that can be recognized by R protein (Drurey *et al*., 2017), and/or the resistance response is suppressed at a later stage (Postma *et al*., 2012; Białas *et al*., 2017; Zhuo *et al*., 2017) (Fig. 4B). Further and more detailed studies are necessary to elucidate the mechanism behind the differential interaction between the two *M. persicae* populations and accession PB2013071.

## Conclusion

Two populations of *M. persicae* (NL and SW) perform similarly with respect to survival and reproduction on two *Capsicum* accessions susceptible to the NL population, but significantly different on three *Capsicum* accessions resistant to that population. The performance difference between the two aphid populations is accompanied by differences in feeding and probing activity as well as in levels of defense response (ROS accumulation, callose deposition), strongly suggesting that the SW population has (partially) overcome the resistance that is effective against the NL population.

## Disclosure

The authors declare that they have no competing interests.

## Supporting information


**Fig. S1**. ROS accumulation in leaves of pepper accession PB2013046 after infestation by *M. persicae* populations NL and SW.

**Table S1**. Evaluation (mean value ± standard deviation) of *Capsicum* accessions for resistance against two *M. persicae* populations NL and SW.

**Table S2**. Proportion of individuals that produced the waveform type (PPW) in EPG recording. Two *M. persicae* populations NL and SW were used for EPG on two pepper accessions PB2013071 and PB2013046.

## References

[ins12648-bib-0001] Alvarez, A. , Tjallingii, W. , Garzo, E. , Vleeshouwers, V. , Dicke, M. and Vosman, B. (2006) Location of resistance factors in the leaves of potato and wild tuber‐bearing Solanum species to the aphid *Myzus persicae* . Entomologia Experimentalis et Applicata, 121, 145–157.

[ins12648-bib-0002] Alvarez, A.E. , Broglia, V.G. , Alberti D'amato, A.M. , Wouters, D. , van Der Vossen, E. , Garzo, E . *et al* (2013) Comparative analysis of *Solanum stoloniferum* responses to probing by the green peach aphid *Myzus persicae* and the potato aphid *Macrosiphum euphorbiae* . Insect Science, 20, 207–227. 23955861 10.1111/j.1744-7917.2012.01505.x

[ins12648-bib-0003] Bass, C. , Puinean, A.M. , Zimmer, C.T. , Denholm, I. , Field, L.M. , Foster, S.P . *et al* (2014) The evolution of insecticide resistance in the peach potato aphid, *Myzus persicae* . Insect Biochemistry and Molecular Biology, 51, 41–51. 24855024 10.1016/j.ibmb.2014.05.003

[ins12648-bib-0004] Białas, A. , Zess, E.K. , De La Concepcion, J.C. , Franceschetti, M. , Pennington, H.G. , Yoshida, K . *et al* (2017) Lessons in effector and NLR biology of plant‐microbe systems. Molecular Plant–Microbe Interactions, 31, 34–45. 29144205 10.1094/MPMI-08-17-0196-FI

[ins12648-bib-0005] Black, L.L. , Green, S.K. , Hartman, G.L. and Poulos, J.M. (1991) Pepper Diseases: A Field Guide, pp. 42–56. Asian Vegetable Research and Development Center.

[ins12648-bib-0006] Blackman, R.L. and Eastop, V.F. (2000) Aphids on the World's Crops: An Identification and Information Guide, pp. 157–158. John Wiley & Sons Ltd.

[ins12648-bib-0007] Bos, J.I. , Prince, D. , Pitino, M. , Maffei, M.E. , Win, J. and Hogenhout, S.A. (2010) A functional genomics approach identifies candidate effectors from the aphid species *Myzus persicae* (green peach aphid). PLoS Genetics, 6, e1001216. 21124944 10.1371/journal.pgen.1001216 PMC2987835

[ins12648-bib-0008] Broekgaarden, C. , Snoeren, T.A. , Dicke, M. and Vosman, B. (2011) Exploiting natural variation to identify insect‐resistance genes. Plant Biotechnology Journal, 9, 819–825. 21679292 10.1111/j.1467-7652.2011.00635.x

[ins12648-bib-0009] Cabrera‐Brandt, M. , Verdugo, J. , Ramirez, C. , Lacroze, J. , Sauge, M. and Figueroa, C. (2015) Intra‐specific variation of behavioral signals in suppressing plant defenses in the green peach aphid *Myzus persicae*, feeding on the resistant wild peach *Prunus davidiana* . Journal of Pest Science, 88, 259–266.

[ins12648-bib-0010] Chandran, P. , Reese, J.C. , Khan, S.A. , Wang, D. , Schapaugh, W. and Campbell, L.R. (2013) Feeding behavior comparison of soybean aphid (Hemiptera: Aphididae) biotypes on different soybean genotypes. Journal of Economic Entomology, 106, 2234–2240. 24224269 10.1603/ec13126

[ins12648-bib-0011] Chaudhary, R. , Atamian, H.S. , Shen, Z. , Briggs, S.P. and Kaloshian, I. (2014) GroEL from the endosymbiont *Buchnera aphidicola* betrays the aphid by triggering plant defense. Proceedings of the National Academy of Sciences USA, 111, 8919–8924. 10.1073/pnas.1407687111 PMC4066539 24927572

[ins12648-bib-0012] Chen, S.X. and Schopfer, P. (1999) Hydroxyl‐radical production in physiological reactions. European Journal of Biochemistry, 260, 726–735. 10103001 10.1046/j.1432-1327.1999.00199.x

[ins12648-bib-0013] Cheng, H.Z. , Zheng, S.Y. , Guo, Y.R. , Zhao, S.Z. and Wang, P. (2004) The resistance of wheat aphids to several insecticides. Henan Agricultural Science, 6, 50–53.

[ins12648-bib-0014] Cleveland, T.E. , Dowd, P.F. , Desjardins, A.E. , Bhatnagar, D. and Cotty, P.J. (2003) United States Department of Agriculture—Agricultural Research Service research on pre‐harvest prevention of mycotoxins and mycotoxigenic fungi in US crops. Pest Management Science, 59, 629–642. 12846313 10.1002/ps.724

[ins12648-bib-0015] Crompton, D. and Ode, P. (2010) Feeding behavior analysis of the soybean aphid (Hemiptera: Aphididae) on resistant soybean “Dowling.” Journal of Economic Entomology, 103, 648–653. 20568609 10.1603/ec09370

[ins12648-bib-0016] Daudi, A. and O'brien, J.A. (2012) Detection of hydrogen peroxide by DAB staining in *Arabidopsis* leaves. Bio‐Protocol, 2, e263. 27390754 PMC4932902

[ins12648-bib-0017] Dixon, A.F.G. (1977) Aphid ecology: life cycles, polymorphism, and population regulation. Annual Review of Ecology and Systematics, 8, 329–353.

[ins12648-bib-0018] Donofrio, N.M. and Delaney, T.P. (2001) Abnormal callose response phenotype and hypersusceptibility to *Peronospora parasitica* in defense‐compromised *Arabidopsis nim1‐1* and salicylate hydroxylase‐expressing plants. Molecular Plant–Microbe Interactions, 14, 439–450. 11310731 10.1094/MPMI.2001.14.4.439

[ins12648-bib-0019] Drurey, C. , Mathers, T.C. , Prince, D.C. , Wilson, C. , Caceres‐Moreno, C. , Mugford, S.T . *et al* (2017) Chemosensory proteins in the CSP4 clade evolved as plant immunity suppressors before two suborders of plant‐feeding hemipteran insects diverged. BioRxiv, 173278.

[ins12648-bib-0020] Eenink, A. , Groenwold, R. and Dieleman, F. (1982) Resistance of lettuce (Lactuca) to the leaf aphid *Nasonovia ribis nigri*. 1. Transfer of resistance from *L. virosa* to *L. sativa* by interspecific crosses and selection of resistant breeding lines. Euphytica, 31, 291–299.

[ins12648-bib-0021] Elzinga, D.A. , De Vos, M. and Jander, G. (2014) Suppression of plant defenses by a *Myzus persicae* (green peach aphid) salivary effector protein. Molecular Plant–Microbe Interactions, 27, 747–756. 24654979 10.1094/MPMI-01-14-0018-R PMC4170801

[ins12648-bib-0022] Flor, H.H. (1971) Current status of the gene‐for‐gene concept. Annual Review of Phytopathology, 9, 275–296.

[ins12648-bib-0023] Furch, A.C. , van Bel, A.J. , Fricker, M.D. , Felle, H.H. , Fuchs, M. and Hafke, J.B. (2009) Sieve element Ca^2+^ channels as relay stations between remote stimuli and sieve tube occlusion in *Vicia faba* . The Plant Cell, 21, 2118–2132. 19602624 10.1105/tpc.108.063107 PMC2729599

[ins12648-bib-0024] Garzo, E. , Fernández‐Pascual, M. , Morcillo, C. , Fereres, A. , Gómez‐Guillamón, M.L. and Tjallingii, W.F. (2018) Ultrastructure of compatible and incompatible interactions in phloem sieve elements during the stylet penetration by cotton aphids in melon. Insect Science, 25, 631–642. 28213963 10.1111/1744-7917.12447

[ins12648-bib-0025] Giordanengo, P. (2014) EPG‐Calc: a PHP‐based script to calculate electrical penetration graph (EPG) parameters. Arthropod–Plant Interactions, 8, 163–169.

[ins12648-bib-0026] Haley, S.D. , Peairs, F.B. , Walker, C.B. , Rudolph, J.B. and Randolph, T.L. (2004) Occurrence of a new Russian wheat aphid biotype in Colorado. Crop Science, 44, 1589–1592.

[ins12648-bib-0027] Hao, P.Y. , Liu, C.X. , Wang, Y.Y. , Chen, R.Z. , Tang, M. , Du, B . *et al* (2008) Herbivore‐induced callose deposition on the sieve plates of rice: an important mechanism for host resistance. Plant Physiology, 146, 1810–1820. 18245456 10.1104/pp.107.111484 PMC2287352

[ins12648-bib-0028] Hebert, S.L. , Jia, L. and Goggin, F.L. (2007) Quantitative differences in aphid virulence and foliar symptom development on tomato plants carrying the *Mi* resistance gene. Environmental Entomology, 36, 458–467. 17445382 10.1603/0046-225x(2007)36[458:qdiava]2.0.co;2

[ins12648-bib-0029] Helden, M. and Tjallingii, W. (1993) Tissue localisation of lettuce resistance to the aphid *Nasonovia ribisnigri* using electrical penetration graphs. Entomologia Experimentalis et Applicata, 68, 269–278.

[ins12648-bib-0030] Hill, C.B. , Crull, L. , Herman, T.K. , Voegtlin, D.J. and Hartman, G.L. (2010) A new soybean aphid (Hemiptera: Aphididae) biotype identified. Journal of Economic Entomology, 103, 509–515. 20429468 10.1603/ec09179

[ins12648-bib-0031] Hogenhout, S.A. and Bos, J.I. (2011) Effector proteins that modulate plant–insect interactions. Current Opinion in Plant Biology, 14, 422–428. 21684190 10.1016/j.pbi.2011.05.003

[ins12648-bib-0032] Humphries, A. , Peck, D. , Robinson, S. , Rowe, T. and Oldach, K. (2013) A new biotype of bluegreen aphid (*Acyrthosiphon kondoi* Shinji) found in south‐eastern Australia overcomes resistance in a broad range of pasture legumes. Crop and Pasture Science, 63, 893–901.

[ins12648-bib-0033] Humphries, A. , Robinson, S. , Hawkey, D. , Peck, D. , Rowe, T. , De Koning, C. *et al* (2016) Diversity for resistance to a moderately virulent bluegreen aphid (*Acyrthosiphon kondoi* Shinji) population in *Trifolium* species. Crop and Pasture Science, 67, 1009–1018.

[ins12648-bib-0034] Jaouannet, M. , Rodriguez, P.A. , Thorpe, P. , Lenoir, C.J. , Macleod, R. , Escudero‐Martinez, C . *et al* (2014) Plant immunity in plant–aphid interactions. Frontiers in Plant Science, 5, 663. 25520727 10.3389/fpls.2014.00663 PMC4249712

[ins12648-bib-0035] Jones, J.D. and Dangl, J.L. (2006) The plant immune system. Nature, 444, 323. 17108957 10.1038/nature05286

[ins12648-bib-0036] Kanvil, S. , Powell, G. and Turnbull, C. (2014) Pea aphid biotype performance on diverse Medicago host genotypes indicates highly specific virulence and resistance functions. Bulletin of Entomolical Research, 104, 689–701. 10.1017/S0007485314000443 25375216

[ins12648-bib-0037] Kenyon, L. , Kumar, S. , Tsai, W.S. and Hughes, J. (2014) Virus diseases of peppers (*Capsicum* spp.) and their control. Advance of Virus Research, 90, 297–354. 10.1016/B978-0-12-801246-8.00006-8 25410105

[ins12648-bib-0038] Kerchev, P.I. , Fenton, B. , Foyer, C.H. and Hancock, R.D. (2012) Infestation of potato (*Solanum tuberosum* L.) by the peach‐potato aphid (*Myzus persicae* Sulzer) alters cellular redox status and is influenced by ascorbate. Plant, Cell & Environment, 35, 430–440. 10.1111/j.1365-3040.2011.02395.x 21736590

[ins12648-bib-0039] Kessler, A. and Baldwin, I.T. (2002) Plant responses to insect herbivory: the emerging molecular analysis. Annual Review of Plant Biology, 53, 299–328. 10.1146/annurev.arplant.53.100301.135207 12221978

[ins12648-bib-0040] Khan, S.A. , Marimuthu, M. , Predeesh, C. , Aguirre‐Rojas, L.M. , Reese, J.C. and Smith, C.M. (2015) Electrical penetration graph recording of Russian wheat aphid (Hemiptera: Aphididae) feeding on aphid‐resistant wheat and barley. Journal of Economic Entomology, 108(5), 2465–2470. 26453736 10.1093/jee/tov183

[ins12648-bib-0041] Kim, K.S. , Hill, C.B. , Hartman, G.L. , Mian, M. and Diers, B.W. (2008) Discovery of soybean aphid biotypes. Crop Science, 48, 923–928.

[ins12648-bib-0042] Kissoudis, C. , Sunarti, S. , van De Wiel, C. , Visser, R.G. , van Der Linden, C.G. and Bai, Y. (2016) Responses to combined abiotic and biotic stress in tomato are governed by stress intensity and resistance mechanism. Journal of Experimental Botany, 67, 5119–5132. 27436279 10.1093/jxb/erw285 PMC5014164

[ins12648-bib-0043] Knoblauch, M. , Froelich, D.R. , Pickard, W.F. and Peters, W.S. (2014) SEORious business: structural proteins in sieve tubes and their involvement in sieve element occlusion. Journal of Experimental Botany, 65, 1879–1893. 24591057 10.1093/jxb/eru071

[ins12648-bib-0044] Kuśnierczyk, A. , Winge, P. , Jørstad, T.S. , Troczyńska, J. , Rossiter, J.T. and Bones, A.M. (2008) Towards global understanding of plant defence against aphids—timing and dynamics of early *Arabidopsis* defence responses to cabbage aphid (*Brevicoryne brassicae*) attack. Plant, Cell & Environment, 31, 1097–1115. 10.1111/j.1365-3040.2008.01823.x 18433442

[ins12648-bib-0045] Lei, J. , Finlayson, S.A. , Salzman, R.A. , Shan, L. and Zhu‐Salzman, K. (2014) *Botrytis*‐induced kinase1 modulates *Arabidopsis* resistance to green peach aphids via phytoalexin deficient4. Plant Physiology, 165, 1657–1670. 24963070 10.1104/pp.114.242206 PMC4119046

[ins12648-bib-0046] Liu, J.L. , Du, H.T. , Ding, X. , Zhou, Y.D. , Xie, P.F. and Wu, J.C. (2017) Mechanisms of callose deposition in rice regulated by exogenous abscisic acid and its involvement in rice resistance to *Nilaparvata lugens* Stål (Hemiptera: Delphacidae). Pest Management Science, 73, 2559–2568. 28664567 10.1002/ps.4655

[ins12648-bib-0047] Liu, X. , Williams, C.E. , Nemacheck, J.A. , Wang, H. , Subramanyam, S. , Zheng, C. and Chen, M.S. (2010) Reactive oxygen species are involved in plant defense against a gall midge. Plant Physiology, 152, 985–999. 19965963 10.1104/pp.109.150656 PMC2815885

[ins12648-bib-0048] Lombaert, E. , Carletto, J. , Piotte, C. , Fauvergue, X. , Lecoq, H. , Vanlerberghe‐Masutti, F. *et al* (2009) Response of the melon aphid, *Aphis gossypii*, to host‐plant resistance: evidence for high adaptive potential despite low genetic variability. Entomologia Experimentalis et Applicata, 133, 46–56.

[ins12648-bib-0049] Luna, E. , Pastor, V. , Robert, J. , Flors, V. , Mauch‐Mani, B. and Ton, J. (2011) Callose deposition: a multifaceted plant defense response. Molecular Plant–Microbe Interactions, 24, 183–193. 20955078 10.1094/MPMI-07-10-0149

[ins12648-bib-0050] Medina‐Ortega, K.J. and Walker, G.P. (2015) Faba bean forisomes can function in defence against generalist aphids. Plant, Cell & Environment, 38, 1167–1177. 10.1111/pce.12470 25311512

[ins12648-bib-0051] Miller, G. , Schlauch, K. , Tam, R. , Cortes, D. , Torres, M.A. , Shulaev, V . *et al* (2009) The plant NADPH oxidase RBOHD mediates rapid systemic signaling in response to diverse stimuli. Science Signaling, 2, ra45–ra45. 19690331 10.1126/scisignal.2000448

[ins12648-bib-0052] Moloi, M.J. and van Der Westhuizen, A.J. (2006) The reactive oxygen species are involved in resistance responses of wheat to the Russian wheat aphid. Journal of Plant Physiology, 163, 1118–1125. 17032617 10.1016/j.jplph.2005.07.014

[ins12648-bib-0053] Pallipparambil, G.R. , Reese, J.C. , Avila, C.A. , Louis, J.M. and Goggin, F.L. (2010) *Mi*‐mediated aphid resistance in tomato: tissue localization and impact on the feeding behavior of two potato aphid clones with differing levels of virulence. Entomologia Experimentalis et Applicata, 135, 295–307.

[ins12648-bib-0054] Peng, H.C. and Walker, G.P. (2018) Sieve element occlusion provides resistance against *Aphis gossypii* in TGR‐1551 melons. Insect Science, 10.1111/1744-7917.12610. PMC7379274 29845727

[ins12648-bib-0055] Peters, W.S. , Van Bel, A.J. and Knoblauch, M. (2006) The geometry of the forisome–sieve element–sieve plate complex in the phloem of *Vicia faba* L. leaflets. Journal of Experimental Botany, 57, 3091–3098. 16882644 10.1093/jxb/erl072

[ins12648-bib-0056] Piedras, P. , Hammond‐Kosack, K.E. , Harrison, K. and Jones, J.D. (1998) Rapid, *Cf*‐9‐and Avr9‐dependent production of active oxygen species in tobacco suspension cultures. Molecular Plant–Microbe Interactions, 11, 1155–1166.

[ins12648-bib-0057] Pitino, M. and Hogenhout, S.A. (2013) Aphid protein effectors promote aphid colonization in a plant species‐specific manner. Molecular Plant–Microbe Interactions, 26, 130–139. 23035913 10.1094/MPMI-07-12-0172-FI

[ins12648-bib-0058] Pitrat, M. and Lecoq, H. (1980) Inheritance of resistance to cucumber mosaic virus transmission by *Aphis gossypii* in *Cucumis* melo. Phytopathology, 70, 958–961.

[ins12648-bib-0059] Postma, W.J. , Slootweg, E.J. , Rehman, S. , Finkers‐Tomczak, A. , Tytgat, T.O. , van Gelderen, K . *et al* (2012) The effector SPRYSEC‐19 of *Globodera rostochiensis* suppresses CC‐NB‐LRR‐mediated disease resistance in plants. Plant Physiology, 160, 944–954. 22904163 10.1104/pp.112.200188 PMC3461567

[ins12648-bib-0060] Powell, G. , Tosh, C.R. and Hardie, J. (2006) Host plant selection by aphids: behavioral, evolutionary, and applied perspectives. Annual Review of Entomology, 51, 309–330. 10.1146/annurev.ento.51.110104.151107 16332214

[ins12648-bib-0061] Rodriguez, P. , Escudero‐Martinez, C. and Bos, J. (2017) An aphid effector targets trafficking protein VPS52 in a host‐specific manner to promote virulence. Plant Physiology, 173, 1892–1903. 10.1104/pp.16.01458 PMC5338666 28100451

[ins12648-bib-0062] Rodriguez, P.A. and Bos, J.I. (2013) Toward understanding the role of aphid effectors in plant infestation. Molecular Plant–Microbe Interactions, 26, 25–30. 23035915 10.1094/MPMI-05-12-0119-FI

[ins12648-bib-0063] Shoala, T. , Edwards, M.G. , Knight, M.R. and Gatehouse, A.M. (2018) OXI1 kinase plays a key role in resistance of Arabidopsis towards aphids (*Myzus persicae*). Transgenic Research, 27, 355–366. 29777502 10.1007/s11248-018-0078-x

[ins12648-bib-0064] Smith, C.M. and Boyko, E.V. (2007) The molecular bases of plant resistance and defense responses to aphid feeding: current status. Entomologia Experimentalis et Applicata, 122, 1–16.

[ins12648-bib-0065] Stewart, H. , Bradshaw, J. and Pande, B. (2003) The effect of the presence of R‐genes for resistance to late blight (*Phytophthora infestans*) of potato (*Solanum tuberosum*) on the underlying level of field resistance. Plant Pathology, 52, 193–198.

[ins12648-bib-0066] Stone, B. and Clarke, A. (1992) Chemistry and Biology of (1, 3)‐D‐glucans. Victoria, Australia, pp. 236–239. La Trobe University Press.

[ins12648-bib-0067] Stotz, H.U. , Kroymann, J. and Mitchell‐Olds, T. (1999) Plant–insect interactions. Current Opinion in Plant Biology, 2, 268–272. 10458997 10.1016/S1369-5266(99)80048-X

[ins12648-bib-0068] Sun, M. , Voorrips, R. , Steenhuis‐Broers, G. , 'van T Westende, W. and Vosman, B. (2018) Reduced phloem uptake of *Myzus persicae* on an aphid resistant pepper accession. BMC Plant Biology, 10.1186/s12870-018-1340-3 PMC6020309 29945550

[ins12648-bib-0069] Tan, M.A. , Hutten, R.C. , Celis, C. , Park, T.‐H. , Niks, R.E. , Visser, R.G . *et al* (2008) The *RPi‐mcd1* locus from *Solanum* microdontum involved in resistance to *Phytophthora infestans*, causing a delay in infection, maps on potato chromosome 4 in a cluster of NBS‐LRR genes. Molecular Plant–Microbe Interactions, 21, 909–918. 18533831 10.1094/MPMI-21-7-0909

[ins12648-bib-0070] ten Broeke, C.J. , Dicke, M. and van Loon, J.J. (2013a) Feeding behaviour and performance of different populations of the black currant‐lettuce aphid, *Nasonovia ribisnigri*, on resistant and susceptible lettuce. Entomologia Experimentalis et Applicata, 148, 130–141.

[ins12648-bib-0071] ten Broeke, C.J. , Dicke, M. and van Loon, J.J. (2013b) Performance and feeding behaviour of two biotypes of the black currant‐lettuce aphid, *Nasonovia ribisnigri*, on resistant and susceptible Lactuca sativa near‐isogenic lines. Bulletin of Entomological Research, 103, 511–521. 23480294 10.1017/S0007485312000880

[ins12648-bib-0072] Tjallingii, W.F. (1988) Electrical recording of stylet penetration activities Aphids: Their Biology, Natural enemies and Control Vol. 2B. (Eds. Minks A.K. & Harewijn P.) pp. 95–108. Elsevier, Amsterdam.

[ins12648-bib-0073] Tjallingii, W.F. (2006) Salivary secretions by aphids interacting with proteins of phloem wound responses. Journal of Experimental Botany, 57, 739–745. 16467410 10.1093/jxb/erj088

[ins12648-bib-0074] Tjallingii, W.F. , Garzo, E. and Fereres, A. (2010) New structure in cell puncture activities by aphid stylets: a dual‐mode EPG study. Entomologia Experimentalis et Applicata, 135, 193–207.

[ins12648-bib-0075] Tolmay, V. , Lindeque, R. and Prinsloo, G. (2007) Preliminary evidence of a resistance‐breaking biotype of the Russian wheat aphid, *Diuraphis noxia* (Kurdjumov) (Homoptera: Aphididae), in South Africa. African Entomology, 15, 228–230.

[ins12648-bib-0076] Tsuda, K. and Katagiri, F. (2010) Comparing signaling mechanisms engaged in pattern‐triggered and effector‐triggered immunity. Current Opinion in Plant Biology, 13, 459–465. 20471306 10.1016/j.pbi.2010.04.006

[ins12648-bib-0077] van der Westhuizen, A. , Qian, X. , Wilding, M. and Botha, A. (2002) Purification and immuno‐cytochemical localization of a wheat β‐1, 3‐glucanase induced by Russian wheat aphid infestation: research letter. South African Journal of Science, 98, 197–202.

[ins12648-bib-0078] van der Westhuizen, A. , Qian, X.M. and Botha, A.M. (1998) β‐1, 3‐Glucanases in wheat and resistance to the Russian wheat aphid. Physiologia Plantarum, 103, 125–131.

[ins12648-bib-0079] Villada, E.S. , González, E.G. , López‐Sesé, A.I. , Castiel, A.F. and Gómez‐Guillamón, M.L. (2009) Hypersensitive response to *Aphis gossypii* Glover in melon genotypes carrying the *Vat* gene. Journal of Experimental Botany, 60, 3269–3277. 19474089 10.1093/jxb/erp163

[ins12648-bib-0080] Wang, K.Y. , Liu, T.‐X. , Yu, C.H. , Jiang, X.Y. and Yi, M.Q. (2002) Resistance of *Aphis gossypii* (Homoptera: Aphididae) to fenvalerate and imidacloprid and activities of detoxification enzymes on cotton and cucumber. Journal of Economic Entomology, 95, 407–413. 12020021 10.1603/0022-0493-95.2.407

[ins12648-bib-0081] Wang, W. , Dai, H. , Zhang, Y. , Chandrasekar, R. , Luo, L. , Hiromasa, Y . *et al* (2015) Armet is an effector protein mediating aphid–plant interactions. The FASEB Journal, 29, 2032–2045. 25678626 10.1096/fj.14-266023

[ins12648-bib-0082] Wu, Z. , Schenk‐Hamlin, D. , Zhan, W. , Ragsdale, D.W. and Heimpel, G.E. (2004) The soybean aphid in China: a historical review. Annals of the Entomological Society of America, 97, 209–218.

[ins12648-bib-0083] Yates, A.D. and Michel, A. (2018) Mechanisms of aphid adaptation to host plant resistance. Current Opinion in Insect Science, 26, 41–49. 29764659 10.1016/j.cois.2018.01.003

[ins12648-bib-0084] Zhuo, K. , Chen, J.S. , Lin, B.R. , Wang, J. , Sun, F.X. , Hu, L.L . *et al* (2017) A novel *Meloidogyne enterolobii* effector MeTCTP promotes parasitism by suppressing programmed cell death in host plants. Molecular Plant Pathology, 18, 45–54. 26808010 10.1111/mpp.12374 PMC6638250

