# Supplementary figures and images for: Aphid populations showing differential levels of virulence on Capsicum accessions
Source: Insect Sci. 2018 Dec 6;27(2):336–48. doi: 10.1111/1744-7917.12648 (PMC7379501; doi:10.1111/1744-7917.12648)

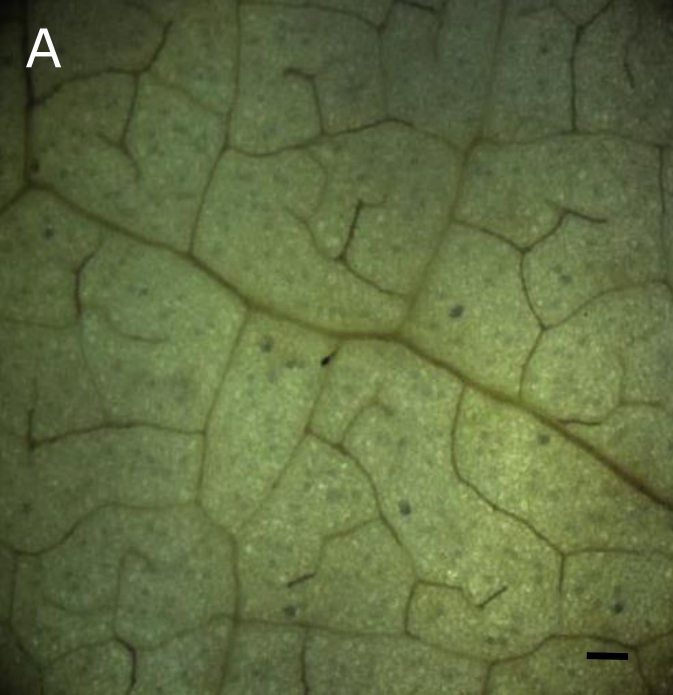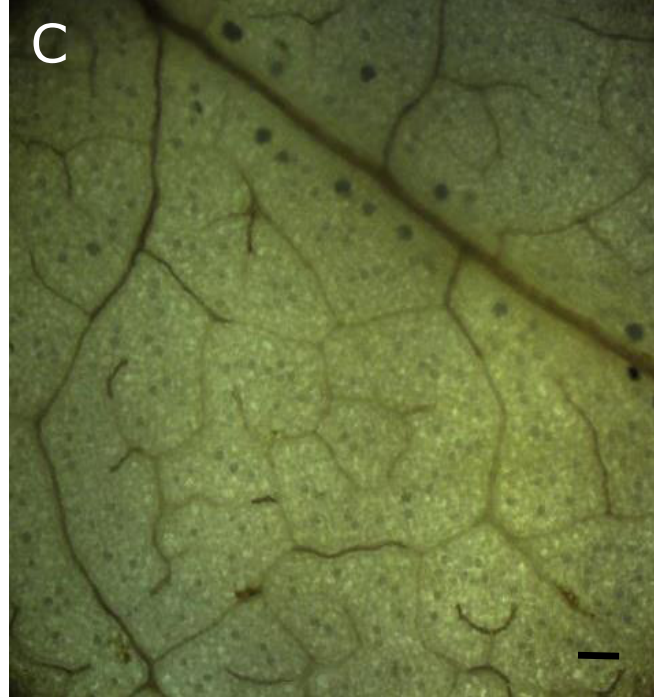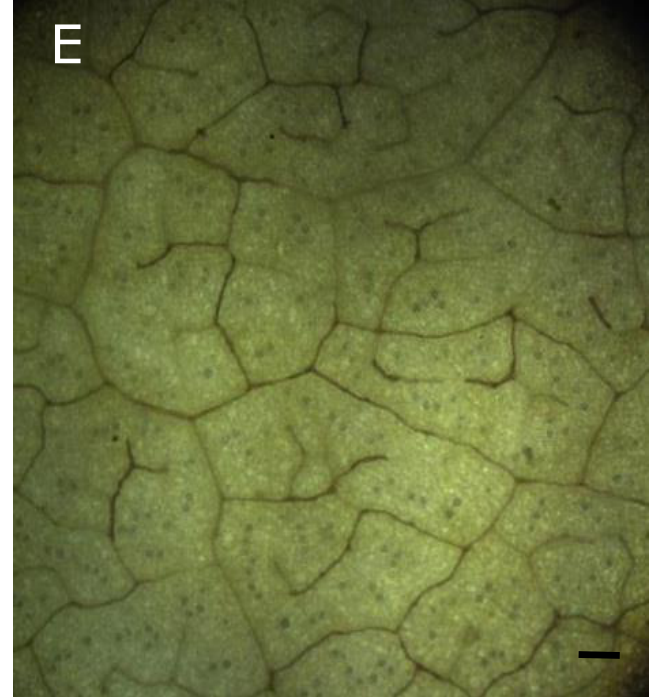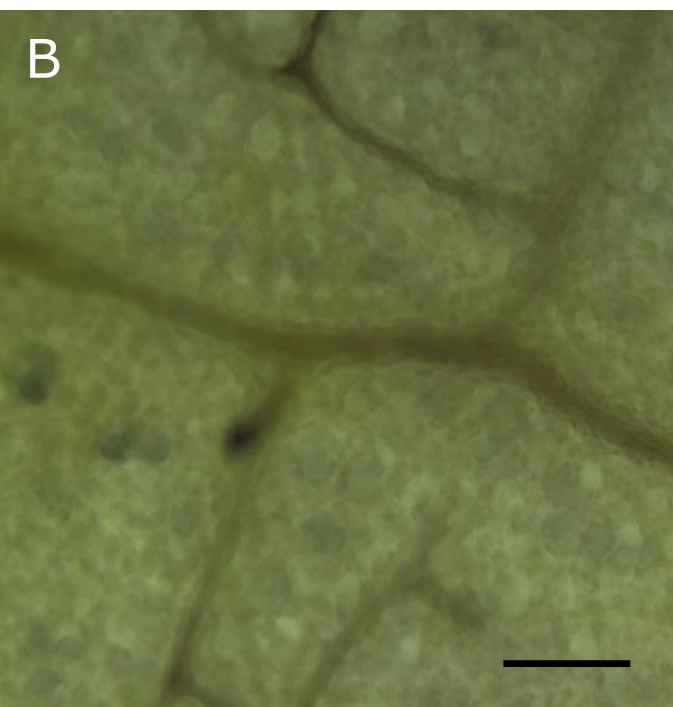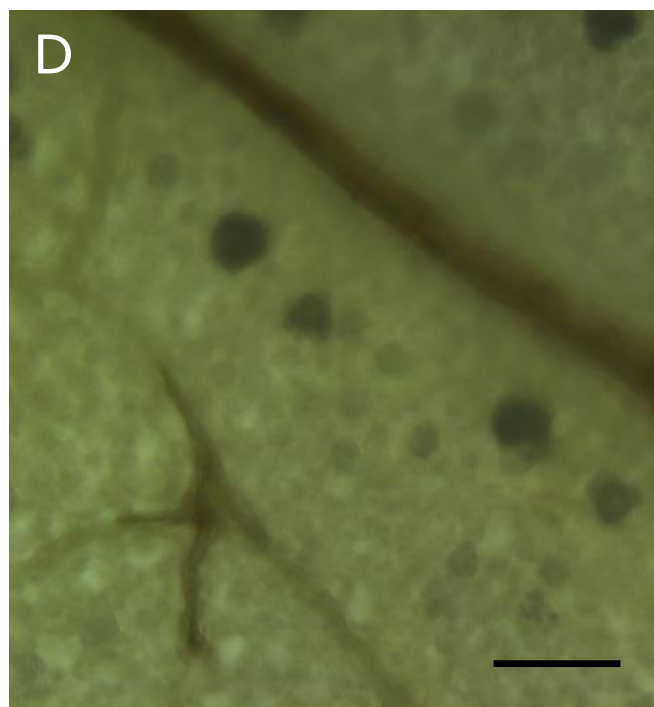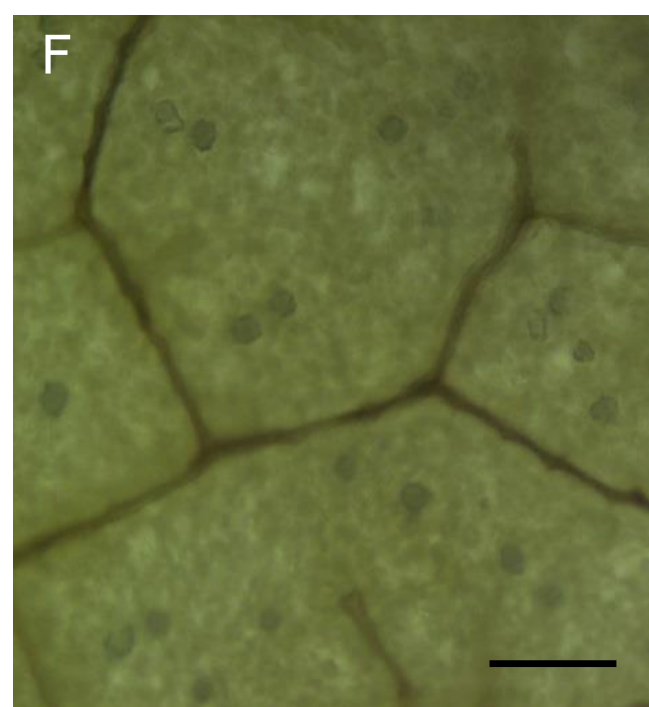

Supplement: Supplementary file 1 — Fig. S1. ROS accumulation in leaves of pepper accession PB2013046 after infestation by M. persicae populations NL and SW. [file INS-27-336-s001.pdf]
