# Supplementary material for: Aphid populations showing differential levels of virulence on Capsicum accessions
Source: Insect Sci. 2018 Dec 6;27(2):336–48. doi: 10.1111/1744-7917.12648 (PMC7379501; doi:10.1111/1744-7917.12648)
Supplement: Supplementary file 2 — Table S1. Evaluation (mean value ± standard deviation) of Capsicum accessions for resistance against two M. persicae populations NL and SW. [file INS-27-336-s002.docx]

**Table S1. Evaluation (mean value ± standard error) of *Capsicum* accessions for resistance against two *M. persicae* populations NL and SW*.***

| **accession** | **Population NL** | | **Population SW** | |
| --- | --- | --- | --- | --- |
|  | **Nymphs ^1^** | **Survival ^2^** | **Nymphs** | **Survival** |
| PB2013046 | 15.08±1.08 | 1.00±0.00 | 12.81±0.53 | 0.99±0.01 |
| CGN19226 | 9.94±0.81 | 0.98±0.02 | 9.61±0.87 | 0.90±0.04 |
| PB2012022 | 1.59±0.39 | 0.42±0.04 | 8.20±0.73 | 0.97±0.02 |
| PB2013062 | 2.53±0.37 | 0.75±0.05 | 10.16±0.43 | 0.97±0.02 |
| PB2013071 | 1.44±0.34 | 0.36±0.08 | 8.35±0.72 | 0.94±0.03 |
| ^1^ Survival refers to fraction of the aphids that survived on an accession after 12 days.  ^2^ Nymphs means average number of next generation nymphs produced per aphid on an accession after 12 days. | | | | |
