# Supplementary material for: Aphid populations showing differential levels of virulence on Capsicum accessions
Source: Insect Sci. 2018 Dec 6;27(2):336–48. doi: 10.1111/1744-7917.12648 (PMC7379501; doi:10.1111/1744-7917.12648)
Supplement: Supplementary file 3 — Table S2. Proportion of individuals that produced the waveform type (PPW) in EPG recording. Two M. persicae populations NL and SW were used for EPG on two pepper accessions PB2013071 and PB2013046. [file INS-27-336-s003.docx]

**Table S2. Proportion of individuals that produced the waveform type (PPW) in EPG recording. Two *M. persicae* populations NL and SW were used for EPG on two pepper accessions PB2013071 and PB2013046.**

| **Waveform type** | **Population NL** | | **Population SW** | |
| --- | --- | --- | --- | --- |
|  | **PB2013071 (n=14)** | **PB2013046 (n=13)** | **PB2013071 (n=14)** | **PB2013046 (n=13)** |
| Probe | 14/14 | 13/13 | 14/14 | 13/13 |
| No-penetration period (NP) | 14/14 | 13/13 | 14/14 | 13/13 |
| Intercellular apoplastic stylet pathway (C) | 14/14 | 13/13 | 14/14 | 13/13 |
| Derailed stylet mechanics (F) | 12/14 | 12/13 | 5/14 | 7/13 |
| Xylem ingestion (G) | 12/14 | 7/14 | 3/14 | 3/13 |
| Phloem phase (E) | 14/14 | 13/13 | 14/14 | 13/13 |
| Phloem salivation (E1) | 14/14 | 13/13 | 14/14 | 13/13 |
| Passive phloem ingestion (E2) | 2/14 | 8/13 | 9/14 | 12/13 |
| Sustained E2 (sE2) | 0/14 | 8/13 | 7/14 | 11/13 |
